# Kindlin-2 inhibits Nlrp3 inflammasome activation in nucleus pulposus to maintain homeostasis of the intervertebral disc

**DOI:** 10.1038/s41413-021-00179-5

**Published:** 2022-01-10

**Authors:** Sheng Chen, Xiaohao Wu, Yumei Lai, Di Chen, Xiaochun Bai, Sheng Liu, Yongchao Wu, Mingjue Chen, Yuxiao Lai, Huiling Cao, Zengwu Shao, Guozhi Xiao

**Affiliations:** 1 Department of Orthopaedics, Union Hospital, Tongji Medical College, Huazhong University of Science and Technology, Wuhan, 430022 China; 2 Department of Biochemistry, School of Medicine, Southern University of Science and Technology, Guangdong Provincial Key Laboratory of Cell Microenvironment and Disease Research, Shenzhen Key Laboratory of Cell Microenvironment, Shenzhen, 518055 China; 3 Department of Orthopedic Surgery, Rush University Medical Center, Chicago, IL 60612 USA; 4 Research Center for Human Tissues and Organs Degeneration, Shenzhen Institutes of Advanced Technology, Chinese Academy of Sciences, Shenzhen, 518055 China; 5 Department of Cell Biology, School of Basic Medical Sciences, Southern Medical University, Guangzhou, 510515 China; 6 Centre for Translational Medicine Research and Development, Institute of Biomedical and Health Engineering, Shenzhen Institute of Advanced Technology, Chinese Academy of Sciences, Shenzhen, 518055 China

**Keywords:** Pathogenesis, Diseases

## Abstract

Intervertebral disc (IVD) degeneration (IVDD) is the main cause of low back pain with major social and economic burdens; however, its underlying molecular mechanisms remain poorly defined. Here we show that the focal adhesion protein Kindlin-2 is highly expressed in the nucleus pulposus (NP), but not in the anulus fibrosus and the cartilaginous endplates, in the IVD tissues. Expression of Kindlin-2 is drastically decreased in NP cells in aged mice and severe IVDD patients. Inducible deletion of Kindlin-2 in NP cells in adult mice causes spontaneous and striking IVDD-like phenotypes in lumbar IVDs and largely accelerates progression of coccygeal IVDD in the presence of abnormal mechanical stress. Kindlin-2 loss activates Nlrp3 inflammasome and stimulates expression of IL-1β in NP cells, which in turn downregulates Kindlin-2. This vicious cycle promotes extracellular matrix (ECM) catabolism and NP cell apoptosis. Furthermore, abnormal mechanical stress reduces expression of Kindlin-2, which exacerbates Nlrp3 inflammasome activation, cell apoptosis, and ECM catabolism in NP cells caused by Kindlin-2 deficiency. In vivo blocking Nlrp3 inflammasome activation prevents IVDD progression induced by Kindlin-2 loss and abnormal mechanical stress. Of translational significance, adeno-associated virus-mediated overexpression of Kindlin-2 inhibits ECM catabolism and cell apoptosis in primary human NP cells in vitro and alleviates coccygeal IVDD progression caused by mechanical stress in rat. Collectively, we establish critical roles of Kindlin-2 in inhibiting Nlrp3 inflammasome activation and maintaining integrity of the IVD homeostasis and define a novel target for the prevention and treatment of IVDD.

## Introduction

Intervertebral disc (IVD) degeneration (IVDD) is one of the main causes of low back pain, which has been estimated as the top reason for years lived with disability globally.^1^ Current management strategies and treatments for IVDD are mainly focused on pain relief and cannot achieve fundamental and long-lasting therapeutic results.^2^ This is in part due to limited understanding of the pathogenesis of IVDD. Better understanding of the underlying pathological mechanisms of IVDD initiation, development and progression will help develop novel strategies for the prevention and treatments for this painful disease.

The IVD is a fibrocartilaginous structure that is located between the upper and lower vertebral bodies of the spine.^3^ IVD is made up of three distinct parts: the central nucleus pulposus (NP), the peripheral annulus fibrosus (AF) and the cartilaginous endplates (CEP) that separate the IVD from the vertebrae. Each part of IVD has distinct cell types, including the NP cells, AF cells, and chondrocytes. These cells synthesize and secret extracellular matrix (ECM) components and play an essential role in maintaining the homeostasis of IVD.^4^ Cumulative evidence suggests that enhanced ECM catabolism and cell apoptosis in NP induced by adverse IVD microenvironments (e.g., abnormal mechanical stress and proinflammatory cytokines) play a vital role in the pathogenesis of IVDD.^5–7^ However, the underlying molecular mechanisms remain poorly understood.

The focal adhesion (FA) protein Kindlin-2, along with other FA proteins such as Talin, Vinculin, activates integrin and regulates several fundamental cellular processes, such as migration and cell-ECM adhesion .^8,9^ Interestingly, recent studies uncover important roles of Kindlin-2 and related proteins, such as Pinch1/2, in control of organogenesis and homeostasis in skeleton^10–13^, kidney,^14,15^ heart,^16,17^ adipose,^18^ pancreas^19^ and intestine through integrin-dependent and independent mechanisms.^20^ Kindlin-2 appears to be extremely critical for skeletal development and homeostasis. For example, deleting Kindlin-2 in Prx1-expressing cells severely impaired both intramembranous and endochondral ossification by inhibiting Tgf-β signaling and Sox9 expression.^10^ Furthermore, Kindlin-2 loss in mature osteoblasts and osteocytes caused severe osteopenia in mice throughout life due to abnormal bone remodeling through upregulation of expression of Sclerostin and Rankl.^11^ In addition, more recent studies have revealed that Kindlin-2 expression in osteocytes mediates the anabolic actions of the intermittent parathyroid hormone in bone^21^ and modulates mechanotransduction in bone.^22^ While above studies clearly confirm the critical requirement for Kindlin-2 in bone and cartilage, whether or not it plays an important role in regulation of IVD homeostasis is unclear.

By utilizing a combination of loss- and gain-of-function approaches at the molecular and histological levels, as well as multiple murine IVDD models, we demonstrate that Kindlin-2 is integral to the IVD homeostasis. Kindlin-2 loss activates NOD-, LRR- and pyrin domain-containing protein 3 (Nlrp3) inflammasome, and accelerates cell apoptosis and ECM catabolism in NP, resulting in spontaneous striking IVDD.

## Results

### Kindlin-2 is highly expressed in NP cells in IVD and drastically reduced in aged mice and severe IVDD patients

**Fig. 1:**
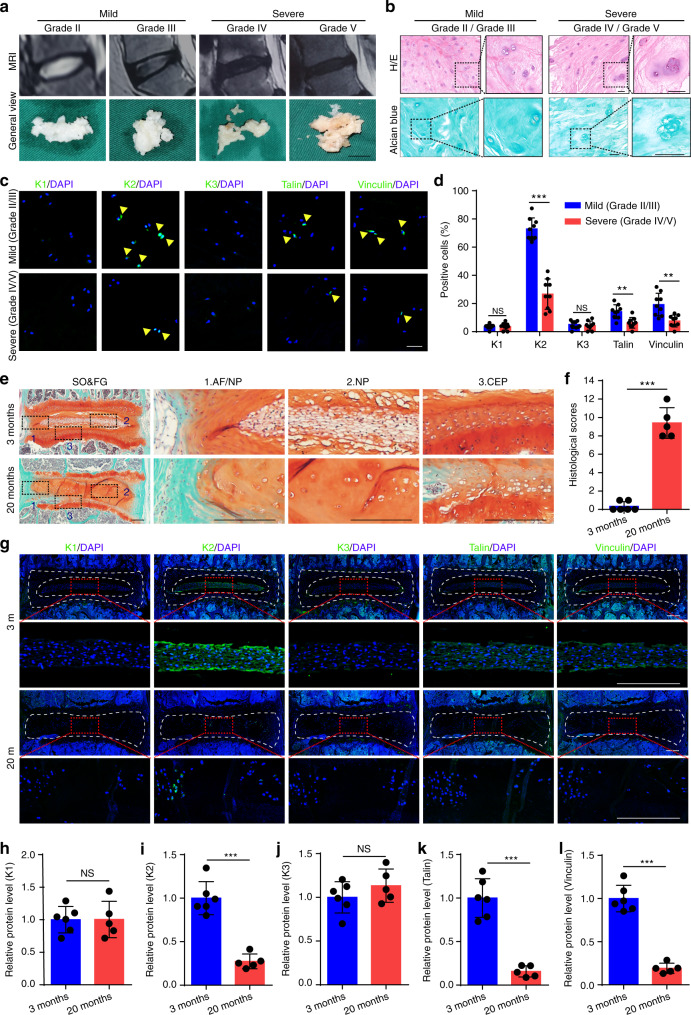
The expression of Kindlin, Talin, and Vinculin proteins in degenerative human nucleus pulposus (NP) specimens and mouse intervertebral discs (IVDs). **a** Representative MRI images and general views of NP tissues with different Pfirrmann degrees. Scale bar, 1 cm. **b** Hematoxylin and eosin (H/E) and Alcian blue staining of human NP samples. Scale bar, 50 μm. **c**, **d** Immunofluorescent (IF) staining of KINDLIN-1, 2, 3 (K1, 2, 3), TALIN, and VINCULIN in human NP samples. Scale bar, 50 μm. *n* = 9. **e**, **f** Safranin O and Fast Green (SO&FG) staining and histological scores of lumbar IVDs in young mice (3-month-old) and aged mice (20-month-old). Scale bar, 200 μm. *n* = 6 (young) and 5 (aged). **g**–**l** IF staining of K1, K2, K3, Talin, and Vinculin in young and aged mice. Scale bar, 200 μm. *n* = 6 (young) and 5 (aged). NS, no statistical significance, **P < 0.01, ***P < 0.001

As an initial attempt to explore the potential role of FA proteins in the pathogenesis of IVDD, we collected a total of 18 degenerative NP samples from IVDD patients and determined the expression of Kindlin-1, Kindlin-2, Kindlin-3, Talin and Vinculin. Pfirrmann grading system, which is based on magnetic resonance imaging (MRI), was first used to grade the degenerative NP specimens (Fig. 1a), and Alcian blue as well as hematoxylin and eosin (H/E) stainings were utilized to further confirm the degenerative degree of the NP samples (Fig. 1b). Immunofluorescent (IF) staining revealed that in human NP tissues, Kindlin-2 was highly expressed. On the contrary, both Kindlin-1 and Kindlin-3 were not expressed in human NP cells. Furthermore, few Vinculin- and Talin- positive cells were detected in human NP (Fig. 1c, d). Importantly, we found that Kindlin-2-positive cells were largely lost in NP tissues from severe IVDD group (Grade IV/V) compared to that from mild IVDD group (Grade II/III) (Fig. 1c, d). Similarly, the numbers of both Talin- and Vinculin-positive cells were obviously decreased in severe versus mild IVDD samples. Results from safranin O and fast green (SO&FG) staining of IVD sections revealed centralized NP mass of vacuolated cells enclosed within a layer of proteoglycan-rich matrix in young (3-month-old) mice (Fig. 1e, top), while loss of clear boundary between the AF and NP and the presence of hypertrophic cells in NP were observed in aged (20-month-old) mice (Fig. 1e, bottom). The histological changes were quantified according to the histological scoring system for mouse IVD.^23^ The histological scores of IVDs in aged mice were significantly higher than in young mice (Fig. 1f). Similar to results from human NP samples, IF staining revealed a strong expression of Kindlin-2, a low expression of Talin and Vinculin and no expression of Kindlin-1 and Kindlin-3 in NP cells in mice (Fig. 1g–l). Furthermore, the expression of Kindlin-2 was drastically decreased in NP tissues from aged versus young mice (Fig. 1g–l).

### Kindlin-2 deletion causes spontaneous and striking IVDD-like phenotypes in lumbar IVDs in mice

**Fig. 2:**
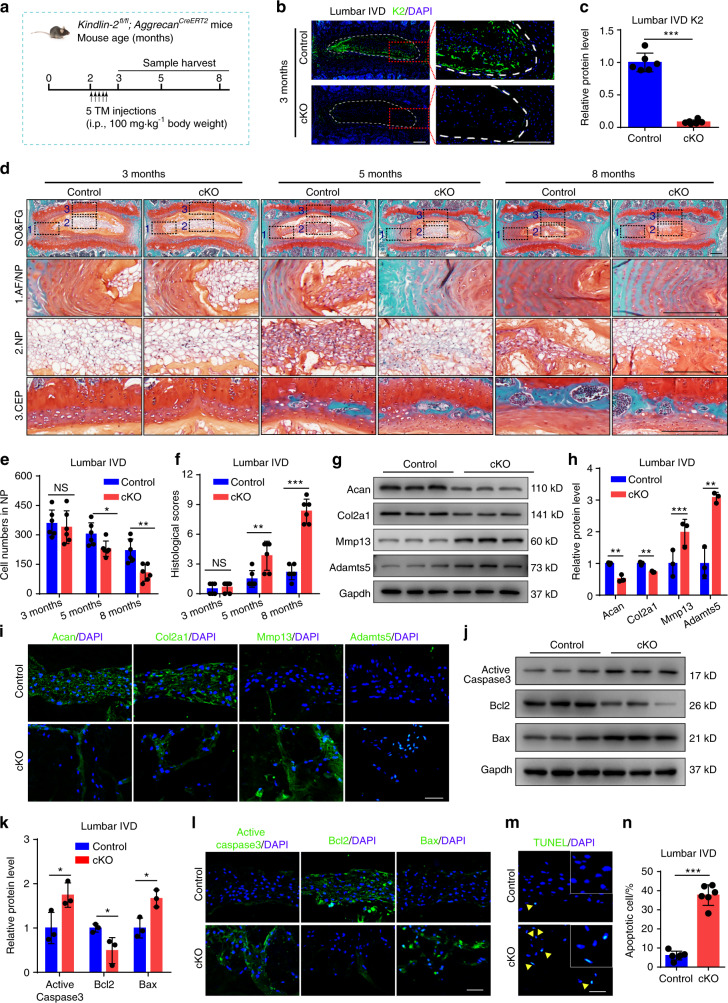
Kindlin-2 deletion results in spontaneous and progressive IVDD in lumbar IVDs in mice. **a** A schematic illustration of the experimental design. **b**, **c** IF staining of K2 in lumbar IVDs in control and Kindlin-2 conditionally knockout (cKO) mice at 3 months of age. Scale bar, 200 μm. *n* = 6. **d**, **f** SO&FG staining and histological scores of lumbar IVDs in control and cKO mice at 3, 5 and 8 months of age. Scale bar, 200 μm. *n* = 6. **e** Cell numbers of NP in control and cKO mice at 3, 5, and 8 months of age. *n* = 6. **g**, **h**, **j**, **k** Western blotting analyses of Aggrecan (Acan), Collagen type II (Col2a1), a disintegrin and metalloproteinase with thrombospondin motif 5 (Adamts5) and matrix metalloproteinase 13 (Mmp13) as well as active Caspase3, Bcl2 and Bax in lumbar IVDs in control and cKO mice at 8 months of age. *n* = 3. **i**, **l** IF staining of Acan, Col2a1, Mmp13 and Adamts5 as well as active Caspase3, Bcl2, and Bax in NP tissues in lumbar IVDs of control and cKO mice at 8 months of age. Scale bar, 50 μm. **m**, **n** TUNEL staining of NP tissues in lumbar IVDs of control and cKO mice at 8 months of age. Scale bar, 50 μm. *n* = 6. NS, no statistical significance, *P < 0.05, **P < 0.01, ***P < 0.001

Above results of Kindlin-2 specific expression in NP cells prompt us to investigate whether Kindlin-2 plays a role in IVD. To this end, we deleted its expression in Aggrecan-expressing cells by treating the two-month-old *Kindlin-2*^*fl/fl*^*; Aggrecan*^*CreERT2*^ male mice with five daily i.p. injections of tamoxifen (TM) (referred to as cKO). Age- and sex-matched mice injected with corn oil were used as controls (Fig. 2a). IF staining indicated that Kindlin-2 was highly expressed in NP cells of lumbar IVD in control mice, which was significantly abolished in NP cells of cKO mice from 1 month after TM injections (Fig. 2b, c, Supplementary Fig. 3a–d). One month after TM injections, cKO mice displayed fairly normal architectures of the lumbar IVD, as revealed by SO&FG staining (Fig. 2d). However, at 3 months after TM injections, apparently less proteoglycan matrix enclosing the NP cells of the lumbar IVD was observed in cKO than that in control mice. Unlike cells in control mice, cKO NP cells did not accomplish a normal morphologic shift from vacuolated and round cells to more flattened cells. Furthermore, loss of clear boundary between the inner AF and NP and the presence of hypertrophic cells in the inner AF were observed in cKO, but not in control mice (Fig. 2d). At 6 months after Kindlin-2 deletion, cell number in the NP area in cKO mice was dramatically decreased, while the remaining cells still exhibited a round and vacuolated morphology (Fig. 2d, e). At all times, no significant differences in outer AF, CEP, and disc height index (DHI) percentage in lumbar IVDs were observed between the two genotypes (Fig. 2d, Supplementary Fig. 3e, f). From 3 months after TM injections, the histological scores in lumbar IVDs were significantly higher in cKO than in control (Fig. 2f).

Disruption of ECM homeostasis and increased cell death are two main characteristics of IVDD. Our results showed that the severity of both features was positively correlated to the progression of IVDD in human NP samples (Supplementary Fig. 4a–f). Results from mice at 6 months after TM injection revealed that Kindlin-2 deletion decreased expression of the anabolic ECM proteins Aggrecan (Acan) and collagen type II (Col2a1) and increased that of the catabolic ECM proteins a disintegrin and metalloproteinase with thrombospondin motif 5 (Adamts5) and matrix metalloproteinase 13 (Mmp13) in lumbar IVDs (Fig. 2g–i). Kindlin-2 deletion promoted NP cell apoptosis in lumbar IVDs (Fig. 2j–n), as demonstrated by increased expression of proapoptotic Bax and active Caspase3 and downregulated expression of the anti-apoptotic Bcl2 as well as increased number of the TUNEL positive cells.

Notably, similar to result from lumbar IVD, Kindlin-2 was highly expressed in the NP cells of coccygeal IVD in control mice, which was drastically decreased in cKO mice from 1 month after TM injection (Supplementary Fig. 5a–c, g). Surprisingly, no remarkable histological changes in coccygeal IVDs were observed between control and cKO groups at all time points (Supplementary Fig. 4d–f). Likewise, there were no significant changes in DHI percentage and histological scores in coccygeal IVDs between the two groups of mice (Supplementary Fig. 4h–j). Collectively, we find that Kindlin-2 loss can cause spontaneous and progressive IVDD in lumbar but not coccygeal IVDs in mice.

### Kindlin-2 deletion accelerates coccygeal IVDD in the presence of abnormal mechanical stress in mice

**Fig. 3:**
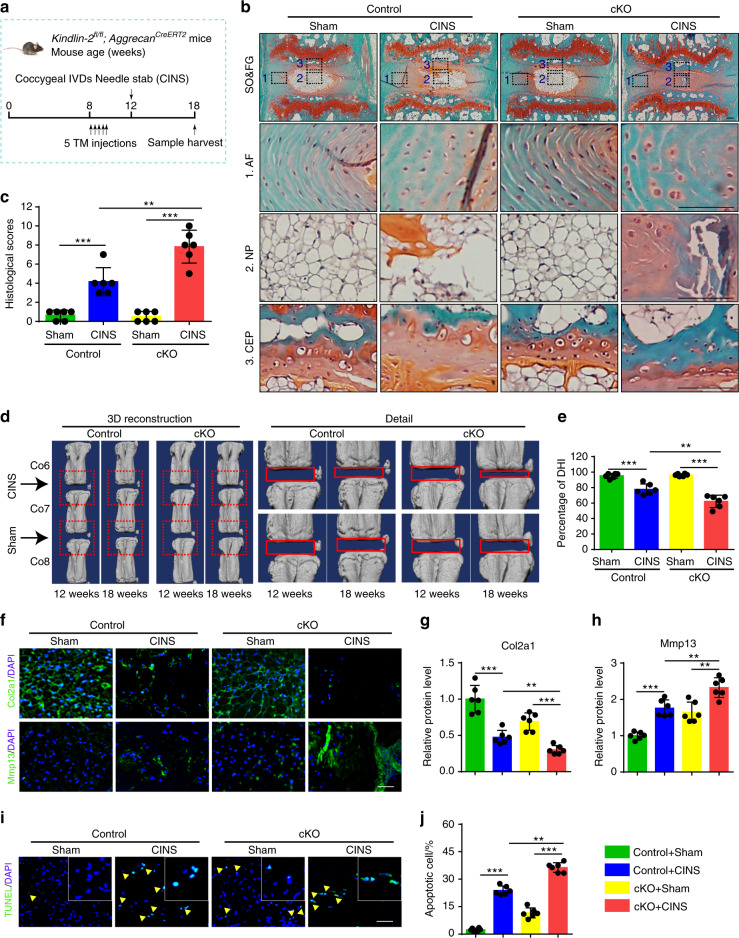
Kindlin-2 deletion accelerates progression of coccygeal IVDD in the presence of abnormal mechanical stress in mice. **a** Overview of the experimental design of coccygeal IVDs needle stab (CINS) model. **b**, **c** SO&FG staining and histological scores of coccygeal IVDs in control and cKO mice with or without CINS. Scale bar, 100 μm. *n* = 6. **d**, **e** Changes in the disc height index (%DHI) (Co6-7, 7–8) were evaluated by micro-computed tomography (μCT) analyses. *n* = 6. **f–h** IF staining of Col2a1 and Mmp13 in NP tissues in coccygeal IVDs of control and cKO mice with or without CINS. Scale bar, 50 μm. *n* = 6. **i**, **j** TUNEL staining of NP tissues in coccygeal IVDs of control and cKO mice with or without CINS. Scale bar, 50 μm. *n* = 6. **P < 0.01, ***P < 0.001

Abnormal mechanical stress is an important contributor of IVDD. In mice, lumbar IVDs bear much greater mechanical stress than coccygeal IVDs do. We wondered whether Kindlin-2 deficiency could promote the degenerative progression of IVDs challenged by abnormal mechanical stress, which may explain above results in that Kindlin-2 deletion caused marked IVDD-like phenotypes in lumbar but not coccygeal IVDs. We next used a coccygeal IVDs needle stab (CINS) model (Fig. 3a), in which the operated coccygeal IVDs were subjected to abnormal mechanical stress due to the altered neutral zone mechanics.^24^ Results from SO&FG staining of coccygeal IVDs in CINS control mice revealed degenerated IVDs characterized by NP clefts, bulging inward inner AF and the presence of hypertrophic AF cells (Fig. 3b). However, more severe degeneration of coccygeal IVDs in CINS cKO mice was observed, as manifested by interrupted border between NP and AF, loss of NP compartment and more hypertrophic AF cells (Fig. 3b). The histologic scores were dramatically increased in CINS cKO mice compared to those in CINS control mice (Fig. 3c). Moreover, micro-computed tomography (μCT) analysis suggested that CINS decreased the DHI percentage, which was exacerbated by Kindlin-2 deletion (Fig. 3d, e). As revealed by IF staining and TUNEL staining, CINS disrupted ECM homeostasis and promoted cell apoptosis in IVDs, which were aggravated by Kindlin-2 deficiency (Fig. 3f–j). Thus, these results indicate that Kindlin-2 deletion accelerates the progression of coccygeal IVDD in the presence of abnormal mechanical stress.

### Abnormal mechanical stress downregulates Kindlin-2, leading to Nlrp3 inflammasome activation, ECM catabolism, and cell apoptosis in NP cells

**Fig. 4:**
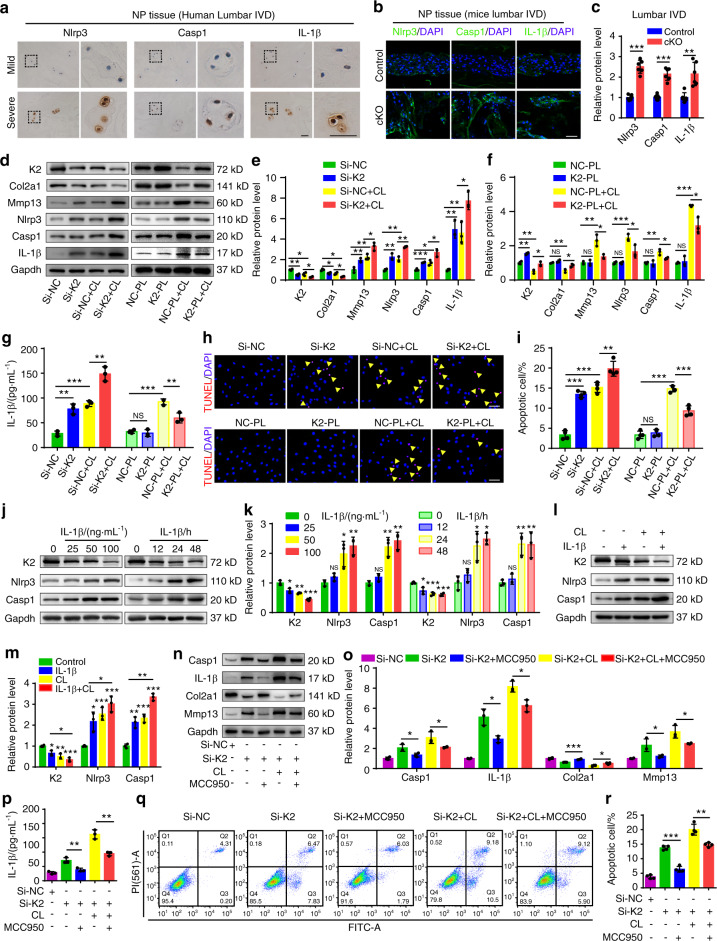
Kindlin-2 deletion promotes Nlrp3 inflammasome activation, ECM catabolism and cell apoptosis in NP cells in vitro. **a** Immunohistochemical (IHC) staining of Nlrp3, Caspase-1(Casp1) and IL-1β in human NP samples. Scale bar, 50 μm. **b**, **c** IF staining of Nlrp3, Casp1 and IL-1β in NP tissues in lumbar IVDs of control and cKO mice at 8 months of age. Scale bar, 50 μm. **d–f** Western blotting analyses of K2, Col2a1, Mmp13, Nlrp3, Casp1, and IL-1β in NP cells, which were transfected with negative control siRNA (si-NC) or K2 siRNA (si-K2), negative control plasmid (NC-PL) or K2 plasmid (K2-PL), and then treated with or without compression loading (CL) treatment. *n* = 3. **g** The levels of IL-1β in the conditioned media of cultured NP cells treated as in (**d**), as detected by ELISA. *n* = 3. **h**, **i** TUNEL staining of NP cells, which were treated as in (d). Scale bar, 50 μm. *n* = 4. **j–m** Western blotting analyses of K2, Nlrp3, and Casp1 in NP cells, which were treated with different doses (0, 25, 50, 100 ng·mL^−1^) of IL-1β for 24 h or 50 ng·mL^−1^ IL-1β for different time (0, 12, 24, 48 h) with or without CL treatment. *n* = 3. **n**, **o** Western blotting analyses of Casp1, IL-1β, Col2a1, and Mmp13 in NP cells, which were transfected with si-NC or si-K2, pretreated with or without MCC950, and then treated with or without CL treatment. *n* = 3. **p** The levels of IL-1β in the conditioned media of cultured NP cells treated as in (**n**), as detected by ELISA. *n* = 3. **q**, **r** Apoptosis rate of NP cells, which were treated as in (**n**), was measured by flow cytometry analyses. *n* = 4. NS no statistical significance, *P < 0.05, **P < 0.01, ***P < 0.001

Previous studies reported that Nlrp3 inflammasome activation plays a key role in promoting IVDD.^25^ In human NP samples, the expression of Nlrp3 inflammasome-related proteins Nlrp3, Caspase-1 (Casp1) and IL-1β was markedly increased in severe IVDD group relative to that in mild IVDD group, as revealed by immunohistochemical (IHC) staining (Fig. 4a). We found that the expression of Nlrp3, Casp1, and IL-1β was upregulated in lumbar IVDs of cKO mice compared to that in control mice (Fig. 4b, c). To further confirm whether Kindlin-2 deletion leads to the Nlrp3 inflammasome activation, we performed Kindlin-2 siRNA knockdown and overexpression experiments in NP cells with and without abnormal compression loading (CL) generated by a customized compression apparatus. Results showed that CL reduced the level of Kindlin-2 protein in NP cells (Fig. 4d, e). Consistent with our in vivo results, Kindlin-2 knockdown decreased the level of Col2a1, increased that of Mmp13 and promoted NP cell apoptosis (Fig. 4d, e, h, i). Consistent with our previously published results,^26^ CL reduced anabolic, but promoted catabolic, protein expression and accelerated apoptosis in NP cells (Fig. 4d, e, h, i). Kindlin-2 knockdown exacerbated the cellular and molecular changes induced by CL (Fig. 4d, e, h, i). In contrast, overexpression of Kindlin-2 alleviated the CL-induced catabolism and cell apoptosis in NP cells (Fig. 4d, f, h, i). Meanwhile, enzyme-linked immunosorbent assay (ELISA) and western blotting analyses showed that Kindlin-2 knockdown or CL activated Nlrp3 inflammasome, as revealed by increased expression of Nlrp3, Casp1, and IL-1β (Fig. 4d–g). Kindlin-2 knockdown further increased the expression levels of Nlrp3, Casp1, IL-1β proteins in CL-treated NP cells, while overexpression of Kindlin-2 suppressed the CL-induced Nlrp3 inflammasome activation (Fig. 4d–g). IL-1β increased the expression of Nlrp3 and Casp1 (Fig. 4j, k), which was previously reported.^25^ More importantly, we found that IL-1β decreased the expression of Kindlin-2 and CL further downregulated Kindlin-2 and upregulated Nlrp3 and Casp1 in IL-1β treated NP cells (Fig. 4j–m). MCC950, a specific inhibitor of Nlrp3 inflammasome activation, blocked basal and CL-induced Nlrp3 inflammasome activation in NP cells with Kindlin-2 knockdown (Fig. 4n–p). Furthermore, MCC950 partially reversed basal and CL-stimulated ECM catabolism and apoptosis in NP cells with Kindlin-2 knockdown (Fig. 4n, o, q, r). Collectively, these results suggest that abnormal mechanical stress stimulates NP cell catabolism and apoptosis probably by promoting the Nlrp3 inflammasome activation through at least in part down-regulation of Kindlin-2.

### Systemic inhibition of Nlrp3 inflammasome activation limits IVDD progression caused by Kindlin-2 deficiency in mice

**Fig. 5:**
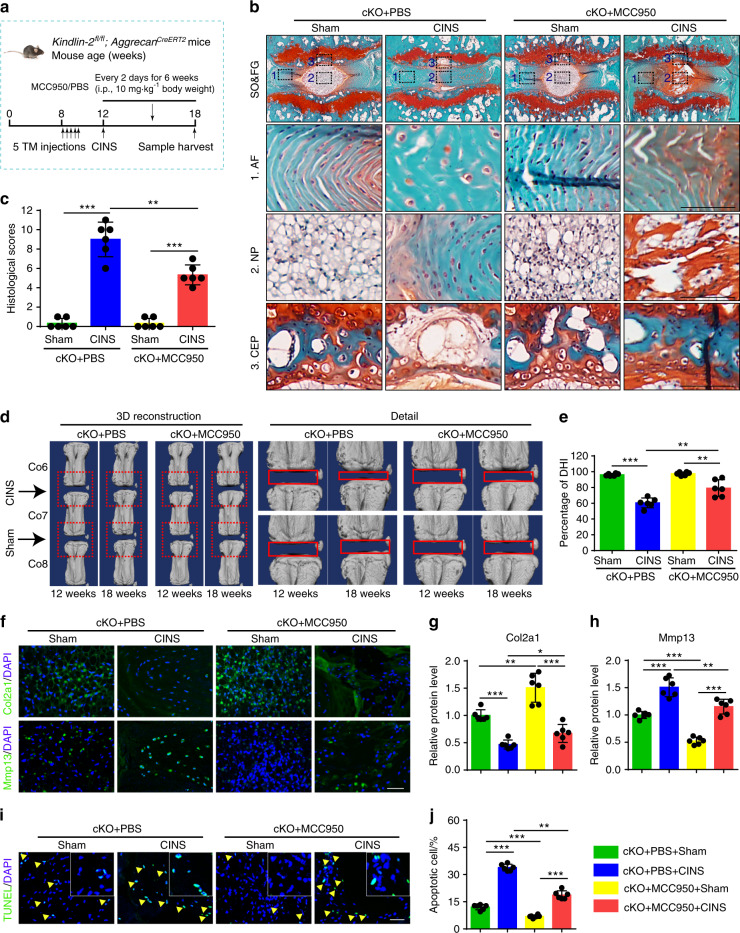
MCC950 limits IVDD progression caused by Kindlin-2 deletion and abnormal mechanical stress in mice. **a** Overview of the experimental set-up of coccygeal IVDs needle stab (CINS) model with or without MCC950 treatment. **b**, **c** SO&FG staining and histological scores of coccygeal IVDs in control and cKO mice, which were treated with or without CINS, and then treated with or without MCC950. Scale bar, 100 μm. *n* = 6. **d**, **e** Changes in the disc height index (%DHI) (Co6-7, 7–8) were evaluated by micro-computed tomography (μCT). *n* = 6. **f–h** IF staining of Col2a1 and Mmp13 in NP tissues in coccygeal IVDs of control and cKO mice treated as in (**b**). Scale bar, 50 μm. *n* = 6. **i**, **j** TUNEL staining of NP tissues in coccygeal IVDs of control and cKO mice treated as in (**b**). Scale bar, 50 μm. *n* = 6. *P < 0.05, **P < 0.01, ***P < 0.001

We next investigated whether pharmacological inhibition of Nlrp3 inflammasome activation can attenuate IVDD in cKO mice with or without CINS. In this experiment, cKO mice with or without CINS were subjected to i.p. injection of MCC950 or PBS as indicated (Fig. 5a). IF staining confirmed that MCC950 essentially abolished the Nlrp3 inflammasome activation (Supplementary Fig. 6a–c). MCC950 ameliorated the progression of coccygeal IVDD and decreased the histologic scores in CINS-treated cKO mice, as revealed by SO&FG staining (Fig. 5b, c). Furthermore, MCC950 improved the DHI percentage in CINS-treated cKO mice, as revealed by μCT analysis (Fig. 5d, e). MCC950 treatment partially alleviated ECM catabolism and increased cell apoptosis in IVDs of CINS cKO mice, as revealed by IF staining and TUNEL staining (Fig. 5f–j). It should be noted that MCC950 treatment also improved ECM catabolism and accelerated cell apoptosis in cKO mice in the absence of CINS (Fig. 5f–j). Moreover, MCC950 treatment alleviated the IVDD-like phenotypes in WT mice with CINS and increased expression of Kindlin-2 protein in NP cells (Supplementary Fig. 7a–d).

### AAV-mediated Kindlin-2 overexpression improves ECM homeostasis and apoptosis in primary human NP cells and alleviates abnormal mechanical stress-induced coccygeal IVDD progression in rat

**Fig. 6:**
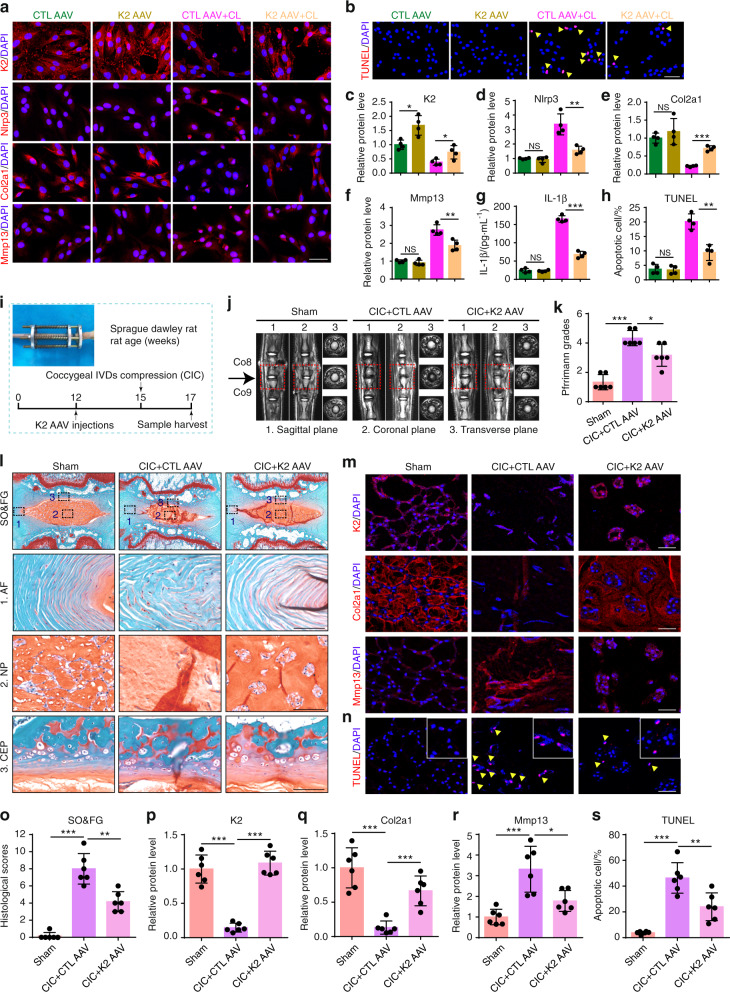
Administration of AAV-Kindlin-2 largely alleviates IVDD progression. **a**, **c–f** IF staining of K2, Nlrp3, Col2a1, and Mmp13 in human primary NP cells infected with control adeno-associated virus (CTL AAV) or K2 AAV, and then treated with or without compression loading (CL). Scale bar, 50 μm. *n* = 4. **g** The levels of IL-1β in the conditioned media of cultured human NP cells treated as in (**a**), as detected by ELISA. *n* = 4. **b**, **h** TUNEL staining of primary human NP cells, which were treated as in (**a**). Scale bar, 50 μm. *n* = 4. **i** Overview of the experimental set-up of rat coccygeal IVDs compression (CIC) model with or without K2 AAV treatment. **j**, **k** Magnetic resonance imaging (MRI) and Pfirrmann grades of coccygeal IVDs in rats treated as in (i). *n* = 6. **l**, **o** SO&FG staining and histological scores of coccygeal IVDs in rats treated as in (**i**). Scale bar, 500 or 100 μm. *n* = 6. **m**, **p-r** IF staining of K2, Col2a1, and Mmp13 in NP tissues in coccygeal IVDs of rats treated as in (**i**). Scale bar, 50 μm. *n* = 6. **n**, **s** TUNEL staining of NP tissues in coccygeal IVDs of rats treated as in (**i**). Scale bar, 50 µm. *n* = 6. *P < 0.05, **P < 0.01, ***P < 0.001

We finally determined whether overexpression of Kindlin-2 impacted IVDD progression by using Kindlin-2-expressing adeno-associated virus (AAV). We first infected human primary NP cells with AAV expressing enhanced green fluorescent protein (AAV EGFP) as control AAV (CTL AAV) or Kindlin-2 AAV (K2 AAV). Results demonstrated a high infection efficiency (Supplementary Fig. 8a, b). Results from IF staining, ELISA analysis, and TUNEL staining showed that Kindlin-2 AAV significantly increased Kindlin-2 expression and inhibited the Nlrp3 inflammasome activation, and protected human NP cells from CL-induced ECM catabolism and cell apoptosis (Fig. 6a–h). We next injected the CTL AAV or Kindlin-2 AAV directly into the rat coccygeal IVDs and applied a coccygeal IVDs compression (CIC) model to induce IVDD (Fig. 6i). Results showed that the strong GFP signal was detected in NP cells at three weeks after AAV injection (Supplementary Fig. 8c, d). Furthermore, administration of Kindlin-2 AAV increased Kindlin-2 expression (Fig. 6m, p), inhibited the Nlrp3 inflammasome activation (Supplementary Fig. 9a–d), and alleviated abnormal mechanical stress-induced coccygeal IVDD, as revealed by improved IVD Pfirrmann grades, histological scores, ECM homeostasis and cell apoptosis (Fig. 6j–s). In addition, AAV-mediated overexpression of Kindlin-2 had no significant effect on IVD structure in rats without loading (Supplementary Fig. 10a–d).

## Discussion

In this study, we for the first time to our knowledge demonstrate an important role of the FA protein Kindlin-2 in NP cells in maintaining the IVD homeostasis. Specifically, we find that Kindlin-2, but not Kindlin-1 and Kindlin-3, is highly expressed in NP cells, but not in AF or CEP cells, in the IVD tissues. We demonstrate that inducible deletion of Kindlin-2 in NP cells in adult mice causes spontaneous striking IVDD phenotypes in lumbar IVDs and largely accelerates the progression of IVDD induced by abnormal mechanical stress in coccygeal IVDs. These important findings uncover a previously unknown role of Kindlin-2 through its expression in NP cells in maintaining integrity of the IVD homeostasis. These results, along with our observations that Kindlin-2 is largely downregulated in NP cells in aged mice and severe IVDD patients, indicate that Kindlin-2 loss may play an essential role in the pathogenesis of IVDD development and progression in humans, which requires further investigation.

While fairly normal architectures of the lumbar IVDs are observed at one month after TM injections, mutant mice start to display marked IVDD phenotypes, which highly mimic those observed in human IVDD patients, at 3 months after TM injections, which becomes worse over time. Kindlin-2 loss dramatically increases the histological scores and decreases DHI percentage in lumbar IVDs at 3 or 6 months after TM injections. At the molecular level, Kindlin-2 loss decreases expression of Acan and Col2a1 and increases that of Mmp13 and Adamts5 and stimulates dramatic ECM catabolism in NP cells. Kindlin-2 loss accelerates NP cell apoptosis by increasing the expression levels of active Caspase3 and Bax proteins and decreasing that of Bcl2 protein. Thus, Kindlin-2 loss impairs the balance of ECM homeostasis and promotes NP cell death in IVDs, two main characteristics of IVDD in humans.^27,28^

We provide several lines of convincing evidence supporting that Kindlin-2 deficiency causes IVDD by promoting Nlrp3 inflammasome activation in NP cells. First, Kindlin-2 loss increases expression of Nlrp3 and IL-1β in NP cells in vitro and in vivo. Second, IL-1β, which is known to activate Nlrp3 inflammasome and plays a key role in the pathogenesis of IVDD,^25^ downregulates Kindlin-2 expression and, in the meantime, increases that of Nlrp3 and Casp1 in dose- and time-dependent manners in NP cells. Third, pharmacological inhibition of Nlrp3 inflammasome activation blocks Kindlin-2 loss induced ECM catabolism and apoptosis in NP cells in vitro and attenuates the progression of IVDD caused by Kindlin-2 loss in mice. Consistent with our findings, recent studies reported that the dysregulated Nlrp3 inflammasome activation is linked to the pathogenesis of other chronic diseases, including neurodegenerative diseases and degenerative osteoarthritis.^29,30^ Furthermore, results from several studies suggest that targeting Nlrp3 inflammasome has a therapeutic effect on IVDD.^25,31^ Results from Tang et al.^32^ demonstrated that honokiol could alleviate oxidative stress-induced ECM degradation and NP cell apoptosis by inhibiting thioredoxin interacting protein/Nlrp3/Casp1/IL-1β signaling axis. Zhao et al^33^ reported that cortistatin could protect against IVDD by suppressing mitochondrial reactive oxygen species (ROS)-dependent activation of the Nlrp3 inflammasome.

It is interesting to note that the IVDD phenotypes caused by Kindlin-2 deficiency are much severe in lumbar IVDs than those in coccygeal IVDs. In fact, at the histological level, the IVDD phenotypes are subtle in coccygeal IVDs in mutant mice even at 3 or 6 months after TM injections, even though marked catabolic gene expression and NP cell apoptosis occur in coccygeal IVDs at these time points. This difference is probably related to the fact that coccygeal IVDs usually bear smaller mechanical force than lumbar IVDs do under physiological conditions.^34^ In support of this notion, we find that, in the presence of abnormal mechanical stress generated via needle stab, Kindlin-2 loss greatly accelerates development and progression of IVDD in coccygeal IVD in adult mice. It is now widely believed that abnormal mechanical stress is one important contributor of IVDD.^35,36^ In human lumbar disc degenerative disease, the majority of spinal disc herniations occur in the lumbar spine at L4-S1 segments, which bear greater mechanical loading than other segments.^37^ Our in vitro gain- and loss-of-function studies demonstrate that either Kindlin-2 knockdown or CL treatment promotes ECM catabolism and cell apoptosis. Furthermore, knockdown of Kindlin-2 exacerbates, while overexpression of Kindlin-2 alleviates, these abnormalities induced by CL treatment. Thus, our results demonstrate that Kindlin-2 loss induces the onset of IVDD and promotes the progression of IVDD under abnormal mechanical stress. Interestingly, we recently reported that Kindlin-2 loss in osteocytes causes significant mechanical property defects and bone loss in weight-bearing long bones, such as ulna, tibia and radius, but not in non-weight-bearing calvarial bones. Furthermore, the loss of Kindlin-2 impairs skeletal responses to mechanical loading stimulation of bone formation in weight-bearing long bones.^22^

In this study, we find that both Kindlin-2 loss or abnormal mechanical stress activates the apoptotic pathway in NP cells, leading to dramatic cell death. In addition, we provide further evidence that Kindlin-2 loss accelerates NP cell apoptosis by activating the IL-1β -Nlrp3 inflammasome axis, which in turn downregulates Kindlin-2, thus creating a vicious cycle in NP cells. First, Kindlin-2 loss increases apoptosis in NP cells, activates Nlrp3 inflammasome, and promotes the release of IL-1β. Second, IL-1β, which is known to induce NP cell apoptosis^38,39^, decreases expression of Kindlin-2 and promotes Nlrp3 inflammasome activation in NP cells, and these changes can be further enhanced by mechanical stress. Importantly, results from the present study show that breaking the vicious cycle by pharmacological inhibition of the Nlrp3 inflammasome activation prevents ECM catabolism, NP cell apoptosis, and IVDD progression induced by Kindlin-2 loss and abnormal mechanical stress.

IVDD is the primary cause of chronic low back pain, which is a public health problem with major social and economic burdens.^40^ Of translational significance, we demonstrate that AAV-mediated expression of Kindlin-2 in IVD tissues benefits ECM homeostasis and inhibits cell apoptosis in human primary NP cells and alleviates coccygeal IVDD progression in rats, thus defining a potential therapeutic target for IVDD.

While, unlike in humans, the NP vacuolar structure in mice remains into adulthood and beyond,^41^ we find that the amount of the NP vacuolar structure is decreased with increased age in control mice, which is delayed in the mutant mice. The underlying molecular mechanism(s) remain to be determined.^42^

It should be pointed out that the expression level of Kindlin-2 protein is extremely low in AF cells in IVDs. While we cannot exclude the possibility that the low Kindlin-2 expression in these cells contributes to the IVDD-like phenotypes in the transgenic mice, this contribution should be very limited. The IVDD-like phenotypes should be primarily due to the deletion of Kindlin-2 in the NP cells, which highly express Kindlin-2 protein.

Our results show that Kindlin-2 protein is almost undetectable in the endplate chondrocytes in adult mice. This may explain why we do not observe any marked phenotypes in the endplate in the mutant mice, although the Cre protein driven by the *Aggrecan* gene promoter may be expressed in these cells.

There are several limitations to this study. First, majority of data from this study were obtained from experiments using small animals, including mice and rats. We plan to investigate the role of Kindlin-2 in IVD in primates in our future study. Second, while our results show that administration of Kindlin-2 AAV by direct injection into IVDs can alleviate abnormal mechanical stress-induced degeneration of rat coccygeal IVDs, the long-term therapeutic effect, and potential side effects are still unclear and need to be studied in the long period experiment. Third, although our results indicate that Kindlin-2 can maintain IVD homeostasis by inhibiting Nlrp3 inflammasome activation in NP cells, detailed molecular mechanisms require further investigation.

In conclusion, we demonstrate that the FA protein Kindlin-2 maintains IVD homeostasis to protect against IVDD by suppressing Nlrp3 inflammasome activation. This work may shed new light on the pathogenesis and therapy of IVDD.

## Materials and methods

### Patient nucleus pulposus (NP) samples

Human NP samples were obtained from 18 patients (8 females and 10 males; mean age 47.9 ± 16.3 years) who were diagnosed with lumbar disc herniation and underwent nucleotomy operations (Supplementary Table 1). MRI-based Pfirrmann grading system was applied to assess the degenerative grade of the NP specimens.^43^ Grade II (*n* = 3)/grade III (*n* = 6) NP specimens were considered as a mild IVD degeneration (IDD) group, and Grade IV (*n*= 5)/grade V (*n* = 4) NP specimens were considered as a severe IDD group.^44^ Ethics approval was obtained from the Ethics Committee of Tongji Medical College, Huazhong University of Science and Technology (No. [2021] IEC (134)). Informed consent was obtained from each participant enrolled in this study.

### Animals

The *Kindlin-2*^*fl/fl*^ mice were developed by our lab as previously described.^10^ The *Aggrecan*^*CreERT2*^ knockin mice were described previously.^45^ To delete Kindlin-2 in aggrecan-expressing cells, the *Kindlin-2*^*fl/fl*^ mice were crossed with *Aggrecan*^*CreERT2*^ mice to get *Kindlin-2*^*fl/+*^*; Aggrecan*^*CreERT2*^ mice, which were then backcrossed with *Kindlin-2*^*fl/fl*^ mice to generate *Kindlin-2*^*fl/fl*^*; Aggrecan*^*CreERT2*^ mice (Supplementary Fig. 1). Two-month-old *Kindlin-2*^*fl/fl*^*; Aggrecan*^*CreERT2*^ male mice were treated with daily i.p. injections of tamoxifen (Sigma-Aldrich; T5648) (1 mg per 10 g body weight) for five days to delete Kindlin-2 expression in aggrecan-positive cells. Sex- and age-matched *Kindlin-2*^*fl/fl*^; *Aggrecan*^*CreERT2*^ mice treated with corn oil (Sigma-Aldrich; C8267) were considered as control mice. We only used male mice for our experiments in order to minimize the use of mice and keep consistency. Mice were group-housed at 20–24 °C and in a cycle of 12-h dark/12-h light. All experimental protocols of animal experiments were approved by the IACUC of SUSTC (No. SUSTC-JY2020119).

### Coccygeal IVDs needle stab IDD model

Two-month-old *Kindlin-2*^*fl/fl*^*; Aggrecan*^*CreERT2*^ mice were subjected with tamoxifen or corn oil via i.p. injection. One month later, CINS IDD model was used to induce IDD as previously described.^24^ Briefly, anesthesia was administered using 2.5% avertin. To locate the position of coccygeal IVDs for needle stab, we performed a small sagittal incision was from Co6 to Co8 in mice tail. Then, we inserted a 31-G needle for 1.5 mm into Co6-7 IVD along vertical direction and parallel to the endplates. Subsequently, the needle was rotated by 180° in the axial direction and held for 10 s. Co7–8 disc was left intact to be a segment of contrast. For IDD experiment, IVDs were obtained at 6 weeks after surgery. For therapeutic experiment, the operated mice were intraperitoneally injected with Nlrp3 inflammasome activation inhibitor MCC950 (Selleck; S7809) at dose of 10 mg/kg body weight or equivalent volume of PBS every 2 days for 6 weeks, then the mice were sacrificed.

### Cell culture and treatments

NP cell line was described in previous studies.^46,47^ The cells were cultured in DMEM with 5% CO_2_ at 37 °C_._ The DMEM contains 10% fetal bovine serum (Gibco; 10099-141) and is supplemented with 1% penicillin–streptomycin (Hyclone; SV30010). We replaced medium every three days and used cells at passage 5–10.

Human primary NP cells were harvested from the part of above patients’ NP samples as previously described.^48^ The obtained NP cells were cultured in DMEM/F-12 (Hyclone; SH30023.01) containing 10% FBS (Gibco; 10099-141) supplemented with 1% penicillin–streptomycin (Hyclone; SV30010) with 5% CO_2_ at 37 °C. We replaced the culture medium every three days and used cells at passage 2.

For CL experiment, a customized compression apparatus (ZL 201120082425.3) was applied. This apparatus provided 1 MPa CL to mimic the abnormal mechanical loading condition in IVD.^36,49,50^ NP cells with or without small interfering RNA (siRNA) transfection, plasmid transfection, Nlrp3 inflammasome activation inhibitor MCC950 treatment (1 μmol·L^−1^; Selleck; S7809) or AAV infection, were seeded on cell culture plates and placed in the compression apparatus for 24 h.

For siRNA transfection, three independent Kindlin-2-siRNAs and a negative control (NC) siRNA (GenePharma) were transfected in NP cells by Lipofectamine^TM^ RNAiMAX Transfection Reagent (50 pmol per 10^5^ cells; ThermoFisher Scientific; 13778150). The most effective target sequence for Kindlin-2-siRNA (#3) was used (Supplementary Fig. 2 and Supplementary Table 2). For plasmid transfection, NC plasmid or Kindlin-2 plasmid (OBIO) were transfected in NP cells by Lipofectamine^TM^ 3000 Transfection Reagent (5 μg per 10^5^ cells; Invitrogen; L3000015). For AAV infection, NP cells were infected with AAV5 expressing enhanced green fluorescent protein (AAV EGFP) as control AAV5 or Kindlin-2 AAV5 (K2 AAV5) (OBIO) at a MOI of 100.

### Western blotting analyses

NP cells and tissues were lysed with RIPA buffer (Sigma-Aldrich; R0278). After centrifugation (13 000 r·min^−^^1^, 4 °C, 10 min), the protein supernatants were isolated from the lysate and used for analysis. Proteins were separated in SDS-PAGE. Then, we blocked membranes using 5% skimmed milk (room temperature, 1 h) after protein transfer, and they were incubated with primary antibody (4 °C, overnight). Next, we washed membranes by TBST (three times) and they were incubated with secondary antibodies (room temperature, 1 h). The enhanced chemiluminescence technique was used to detect the bands. Antibody information are listed in Supplementary Table 3.

### Histology and immunostaining assays

Human NP samples were fixed for 48 h using 4% paraformaldehyde (PFA), dehydrated, paraffin-embedded, and sectioned at 5 μm. The sections were stained by Alcian blue and H/E. Mice IVDs (L4-5 lumbar discs, Co6-7, 7–8 coccygeal discs) were fixed for 48 h in 4% PFA, decalcified for 14 days with 10% ethylenediaminetetraacetic acid (EDTA; pH 7.2), dehydrated, paraffin embedded and sectioned at 5 μm before staining. Rat IVDs (Co8-9 coccygeal discs) were fixed for 48 h in 4% PFA, decalcified for 8 weeks with 10% EDTA, dehydrated, paraffin embedded and sectioned at 5 μm. The prepared sections were stained by safranin O and fast green (SO&FG). The histological scores of IVDs were assessed according to histological scoring system for IVD as previously described.^23^

For IHC staining, sections were deparaffinized by xylene, and then they were rehydrated using ethanol. Citrate buffer (0.1 mol·L^−1^, pH 6.0) was used to perform antigen-retrieval. After blocked using peroxidase-blocking solution and normal horse serum, sections were incubated with primary antibodies (4 °C, overnight). Then, sections were incubated with biotinylated IgG and streptavidin-horseradish peroxidase. DAB Peroxidase Substrate Kit was used to visualize the immunoreactivity. Finally, sections were counterstained by hematoxylin, and then mounted. Antibody information are listed in Supplementary Table 3.

For IF staining, sections were prepared in the same way of IHC staining. After blocked with QuickBlock™ Blocking Buffer (Beyotime) added with Triton 100 (Sigma-Aldrich), sections were incubated with primary antibodies (4°C, overnight). Then, sections were incubated with anti-mouse/rabbit Alexa Fluor 488 or 568 secondary antibodies. Finally, sections were examined by confocal microscope and evaluated using ImageJ software. Antibody information are listed in Supplementary Table 3.

### Micro-Computed Tomography (μCT) analysis

After anesthesia with 2.5% avertin, the lumbar and caudal spine of mice was scanned with source voltage of 60 kV and current of 100 μA resulting in 18 μm image pixel size by the high-resolution μCT scanner (Bruker, Skyscan1276). IVD height and the length of the adjacent vertebral body (L4-5, Co6-7, 7–8) were obtained by measuring the midline and the lines of 1/4 IVD’s width from the midline on both sides. The DHI was calculated by the mean of the 3 measurements of IVD height divided by the length of adjacent vertebral body. DHI change (%DHI) was calculated by a percentage of post-DHI/pre-DHI as previously described.^51^

### Apoptosis analysis

TUNEL staining was used to analyze cell apoptosis. For NP cells seeded in the culture plate, after fixed for 15 min in 4% PFA, cells were permeabilized for 10 min using 0.1% TritonX-100. Next, the cells were washed by PBS and stained by TUNEL staining (Beyotime; C1088, C1090) at 37 °C in the dark for 1 h. For paraffin sections, after the processes of deparaffinization and rehydration, sections were incubated using protein Kinase (20 μg·mL^−1^) for 15 min at 37 °C. Then, the sections were washed by PBS and stained with TUNEL staining. Finally, apoptotic cells were assessed by the confocal microscope (A1R; Nikon). The apoptosis rate of NP cells was evaluated by Annexin V-FITC/PI Apoptosis Detection Kit (Beyotime; C1062). Briefly, after trypsinization, NP cells were resuspended in 500 μL binding buffer, followed by the addition of 5 μL Annexin V-FITC and PI. After incubated in the dark for 10 min at room temperature, the apoptosis rate was analyzed by FACSCanto Analyzer (BD Biosciences).

### Enzyme-linked immunosorbent assay (ELISA)

The conditioned media of cultured NP cells with different treatments were stored at −80 °C in a freezer until detection. The levels of interleukin (IL)-1β were measured using the rat IL-1β ELISA Kit (ABclonal; RK00009) or human IL-1β ELISA Kit (ABclonal; RK00001) following the manufacturer’s instructions.

### Coccygeal IVDs compression model

Three-month-old Sprague Dawley rats were administrated with control AAV5 or K2 AAV5 (3.2 × 10^10^ particles in 2 μL) by direct injection into the rat coccygeal IVDs with a 33-gauge needle (Hamilton, Switzerland). Three weeks later, the CIC IDD model was used to induce IDD as previously described^35,50^. Briefly, anesthesia was administered using isoflurane (RWD; R510-22-4). Then, C8 and C9 caudal vertebras were attached by carbon fiber rings, through 0.8-mm Kirschner wires. Axial loading was applied using four 0.50-N·mm^−1^ calibrated springs installed over each rod. The compressive loading provided by the device is 1.3 MPa. Sham animals were administrated with PBS (2 μL) by direct injection into the coccygeal IVDs 3 weeks before the surgery, and also attached with the loading device, but no compressive pressure was exerted onto the IVDs. The rats were examined with MRI (United Imaging; uMR790) and sacrificed at 2 weeks after the surgery.

### Statistical analysis

Data were presented as mean ± standard deviation (SD) from at least three independent experiments. Unpaired student’s *t* tests were applied in the analysis of two-group parameters. One-way analysis of variance was used in comparisons of multiple groups, followed by Tukey’s post hoc test. All statistical analyses were carried out with GraphPad Prism 6 software (GraphPad Software Inc.). *P* < 0.05 was considered statistically significant.

## Supplementary information

Supplementary Materials

## Data Availability

All data are available from the corresponding authors upon reasonable request.

## Patient and public involvement

Human nucleus pulposus samples were obtained from patients who were diagnosed with lumbar disc herniation and underwent nucleotomy operations, following informed written patient consent with approval from The Ethics Committee of Tongji Medical College, Huazhong University of Science and Technology (No. [2021] IEC (134)).

## References

[1] Vos T (2020). Global burden of 369 diseases and injuries in 204 countries and territories, 1990–2019: a systematic analysis for the Global Burden of Disease Study 2019. Lancet.

[2] Clouet J (2019). Intervertebral disc regeneration: from cell therapy to the development of novel bioinspired endogenous repair strategies. Adv. Drug Deliv. Rev..

[3] Lyu FJ (2019). IVD progenitor cells: a new horizon for understanding disc homeostasis and repair. Nat. Rev. Rheumatol..

[4] Frapin L (2019). Lessons learned from intervertebral disc pathophysiology to guide rational design of sequential delivery systems for therapeutic biological factors. Adv. Drug Deliv. Rev..

[5] Ma K (2019). Mechanisms of endogenous repair failure during intervertebral disc degeneration. Osteoarthr. Cartil..

[6] Risbud MV, Shapiro IM (2014). Role of cytokines in intervertebral disc degeneration: pain and disc content. Nat. Rev. Rheumatol..

[7] Vergroesen PP (2015). Mechanics and biology in intervertebral disc degeneration: a vicious circle. Osteoarthr. Cartil..

[8] Sun Z, Costell M, Fassler R (2019). Integrin activation by talin, kindlin and mechanical forces. Nat. Cell Biol..

[9] Plow EF, Qin J (2019). The kindlin family of adapter proteins. Circ. Res..

[10] Wu C (2015). Kindlin-2 controls TGF-beta signalling and Sox9 expression to regulate chondrogenesis. Nat. Commun..

[11] Cao H (2020). Focal adhesion protein Kindlin-2 regulates bone homeostasis in mice. Bone Res.

[12] Lei Y (2020). LIM domain proteins Pinch1/2 regulate chondrogenesis and bone mass in mice. Bone Res.

[13] Wang Y (2019). Focal adhesion proteins Pinch1 and Pinch2 regulate bone homeostasis in mice. JCI Insight.

[14] Sun Y (2017). Kindlin-2 association with rho GDP-dissociation inhibitor alpha suppresses Rac1 activation and podocyte injury. J. Am. Soc. Nephrol..

[15] Wei X (2013). Kindlin-2 mediates activation of TGF-beta/Smad signaling and renal fibrosis. J. Am. Soc. Nephrol..

[16] Zhang Z (2019). Kindlin-2 is essential for preserving integrity of the developing heart and preventing ventricular rupture. Circulation.

[17] Qi L (2019). Kindlin-2 suppresses transcription factor GATA4 through interaction with SUV39H1 to attenuate hypertrophy. Cell Death Dis..

[18] Gao H (2019). Lipoatrophy and metabolic disturbance in mice with adipose-specific deletion of kindlin-2. JCI Insight.

[19] Zhu K (2020). Kindlin-2 modulates MafA and beta-catenin expression to regulate beta-cell function and mass in mice. Nat. Commun..

[20] He X (2020). Kindlin-2 deficiency induces fatal intestinal obstruction in mice. Theranostics.

[21] Fu X (2020). Kindlin-2 regulates skeletal homeostasis by modulating PTH1R in mice. Signal Transduct. Target Ther..

[22] Qin L (2021). Kindlin-2 mediates mechanotransduction in bone by regulating expression of Sclerostin in osteocytes. Commun. Biol..

[23] Tam V (2018). Histological and reference system for the analysis of mouse intervertebral disc. J. Orthop. Res.

[24] Ji ML (2018). Preclinical development of a microRNA-based therapy for intervertebral disc degeneration. Nat. Commun..

[25] Chen F (2020). Melatonin alleviates intervertebral disc degeneration by disrupting the IL-1beta/NF-kappaB-NLRP3 inflammasome positive feedback loop. Bone Res..

[26] Lin H (2017). Edaravone ameliorates compression-induced damage in rat nucleus pulposus cells. Life Sci..

[27] Yang S (2020). Intervertebral disc ageing and degeneration: the antiapoptotic effect of oestrogen. Ageing Res Rev..

[28] Chen S (2019). TGF-beta signaling in intervertebral disc health and disease. Osteoarthr. Cartil..

[29] Mangan MSJ (2018). Targeting the NLRP3 inflammasome in inflammatory diseases. Nat. Rev. Drug Disco..

[30] Swanson KV, Deng M, Ting JP (2019). The NLRP3 inflammasome: molecular activation and regulation to therapeutics. Nat. Rev. Immunol..

[31] Hong J (2020). Bromodomain-containing protein 4 inhibition alleviates matrix degradation by enhancing autophagy and suppressing NLRP3 inflammasome activity in NP cells. J. Cell Physiol..

[32] Tang P (2018). Honokiol alleviates the degeneration of intervertebral disc via suppressing the activation of TXNIP-NLRP3 inflammasome signal pathway. Free Radic. Biol. Med..

[33] Zhao Y (2020). Cortistatin protects against intervertebral disc degeneration through targeting mitochondrial ROS-dependent NLRP3 inflammasome activation. Theranostics.

[34] Holguin N (2014). The aging mouse partially models the aging human spine: lumbar and coccygeal disc height, composition, mechanical properties, and Wnt signaling in young and old mice. J. Appl. Physiol. (1985).

[35] Bian Q (2017). Mechanosignaling activation of TGFbeta maintains intervertebral disc homeostasis. Bone Res..

[36] Chen S (2017). Mesenchymal stem cells protect nucleus pulposus cells from compression-induced apoptosis by inhibiting the mitochondrial pathway. Stem Cells Int..

[37] Saleem S (2013). Lumbar disc degenerative disease: disc degeneration symptoms and magnetic resonance image findings. Asian Spine J..

[38] Wang K (2019). Ligustilide alleviated IL-1β induced apoptosis and extracellular matrix degradation of nucleus pulposus cells and attenuates intervertebral disc degeneration in vivo. Int. Immunopharmacol..

[39] Cheng X (2018). Circular RNA VMA21 protects against intervertebral disc degeneration through targeting miR-200c and X linked inhibitor-of-apoptosis protein. Ann. Rheum. Dis..

[40] Wu A (2020). Global low back pain prevalence and years lived with disability from 1990 to 2017: estimates from the Global Burden of Disease Study 2017. Ann. Transl. Med..

[41] Roberts S (2002). Disc morphology in health and disease. Biochem Soc. Trans..

[42] Rodrigues-Pinto R, Richardson SM, Hoyland JA (2014). An understanding of intervertebral disc development, maturation and cell phenotype provides clues to direct cell-based tissue regeneration therapies for disc degeneration. Eur. Spine J..

[43] Pfirrmann CW (2001). Magnetic resonance classification of lumbar intervertebral disc degeneration. Spine (Philos. Pa 1976).

[44] Hu B (2016). Heme oxygenase-1 attenuates IL-1beta induced alteration of anabolic and catabolic activities in intervertebral disc degeneration. Sci. Rep..

[45] Henry SP (2009). Generation of aggrecan-CreERT2 knockin mice for inducible Cre activity in adult cartilage. Genesis.

[46] Oh CD (2016). Rho-associated kinase inhibitor immortalizes rat nucleus pulposus and annulus fibrosus cells: establishment of intervertebral disc cell lines with novel approaches. Spine (Philos. Pa 1976).

[47] Chen S (2020). Moderate fluid shear stress regulates heme oxygenase-1 expression to promote autophagy and ECM homeostasis in the nucleus pulposus cells. Front Cell Dev. Biol..

[48] Liao Z (2019). Exosomes from mesenchymal stem cells modulate endoplasmic reticulum stress to protect against nucleus pulposus cell death and ameliorate intervertebral disc degeneration in vivo. Theranostics.

[49] Ma KG (2013). Autophagy is activated in compression-induced cell degeneration and is mediated by reactive oxygen species in nucleus pulposus cells exposed to compression. Osteoarthr. Cartil..

[50] He R (2021). HIF1A Alleviates compression-induced apoptosis of nucleus pulposus derived stem cells via upregulating autophagy. Autophagy.

[51] Han B (2008). A simple disc degeneration model induced by percutaneous needle puncture in the rat tail. Spine (Philos. Pa 1976).

