# Supplementary material for: Kindlin-2 inhibits Nlrp3 inflammasome activation in nucleus pulposus to maintain homeostasis of the intervertebral disc
Source: Bone Res. 2022 Jan 10;10:5. doi: 10.1038/s41413-021-00179-5 (PMC8748798; doi:10.1038/s41413-021-00179-5)
Supplement: Supplementary file 1 — Supplementary Materials [file 41413_2021_179_MOESM1_ESM.docx]

**Supplementary Figures**

**
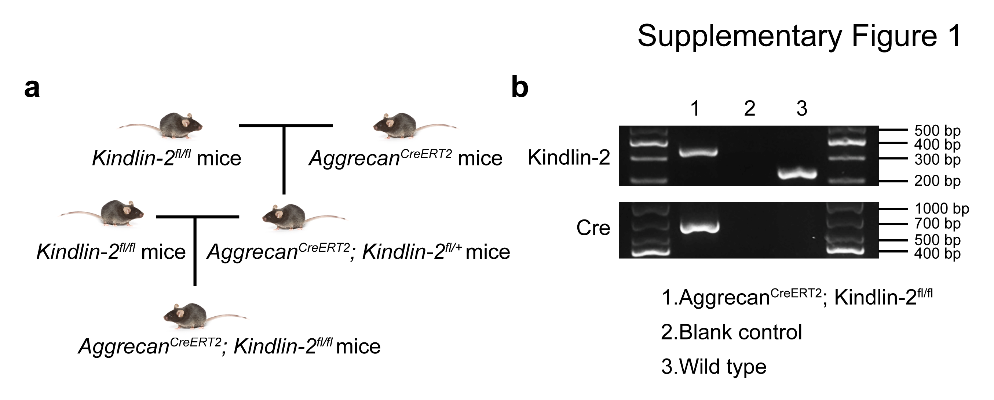
**

**Supplementary Figure 1.** **Breeding strategy and genotyping.** **(a)** Breeding strategy. **(b)** Genotyping using tail DNA. Kindlin-2 (K2) floxed band: ~300bp; Aggrecan^CreERT2^ band: ~650bp. Primer sequences used in PCR genotyping are listed in Supplementary Table 2.


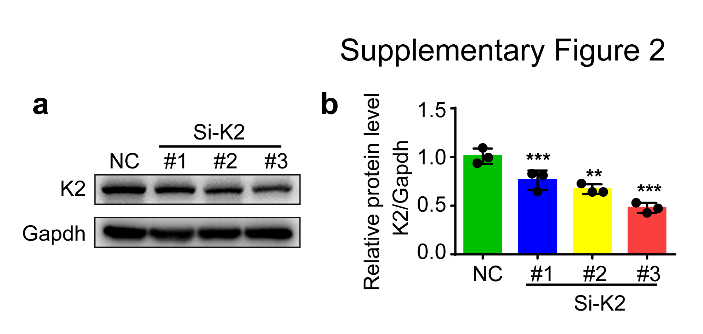


**Supplementary Figure 2.** **The most effective target sequence for Kindlin-2-siRNA was #3.** **(a, b)** Western blotting analyses of K2 in nucleus pulposus (NP) cells transfected with negative control (NC) siRNA and three independent Kindlin-2-siRNAs. *N* = 3. Results are expressed as mean ± standard deviation (s.d.). ***P* < 0.01, ****P* < 0.001.


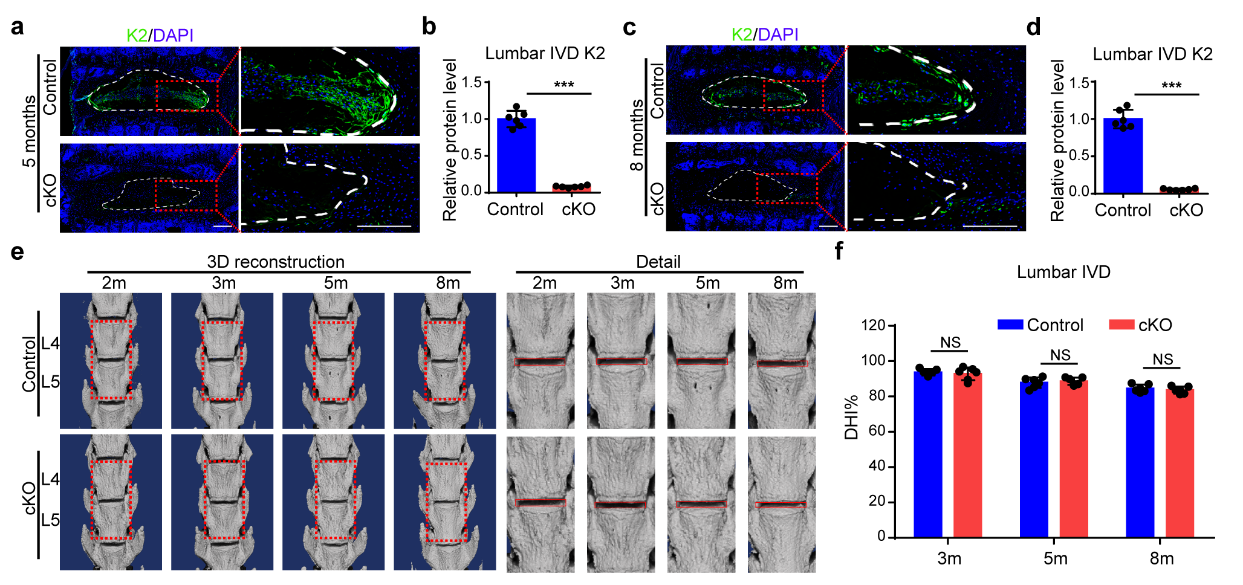


**Supplementary Figure 3.** **Kindlin-2 deletion in lumbar** **intervertebral discs.** **(a, b)** Immunofluorescent (IF) staining of K2 in lumbar intervertebral discs (IVDs) in control and cKO mice at 5 months of age. bar, 200 μm. *N* = 6. **(c, d)** IF staining of K2 in lumbar IVDs in control and cKO mice at 8 months of age. bar, 200 μm. *N* = 6. **(e, f)** Disc height index percentage (%DHI) of lumbar IVDs (L4-5) of the control and cKO mice was evaluated by micro-computed tomography (μCT). *N* = 6. Results are expressed as mean ± standard deviation (s.d.). NS, no statistical significance, ****P* < 0.001.


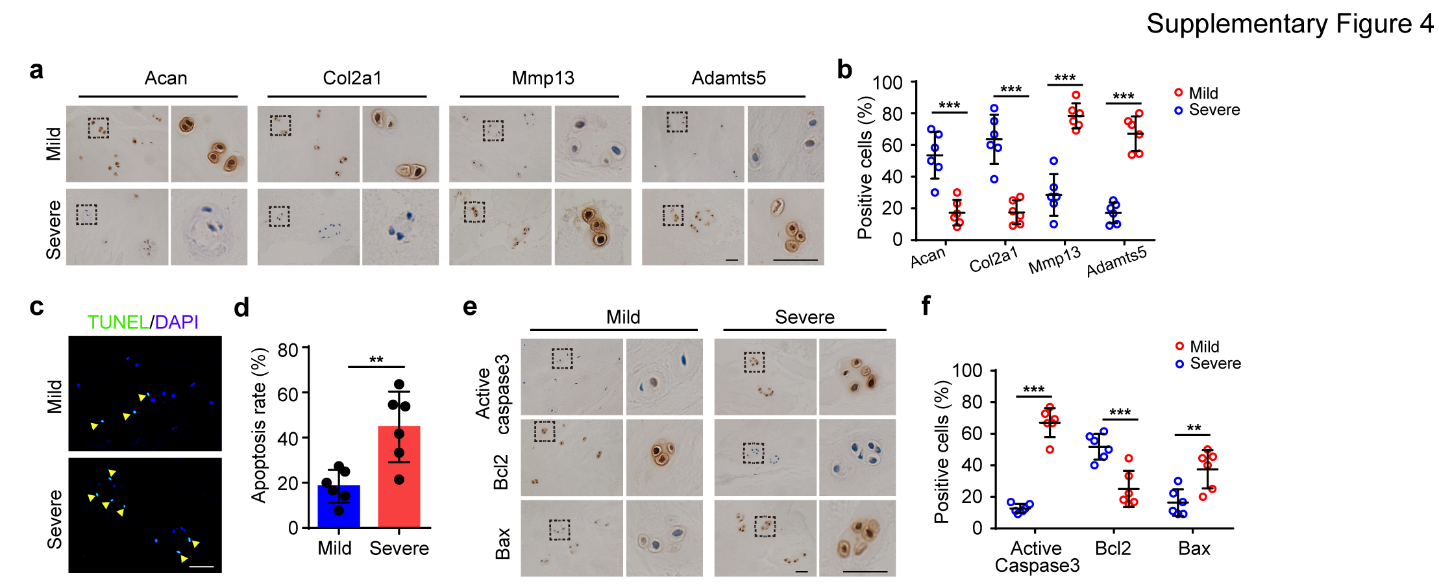


**Supplementary Figure 4.** **Increased cell death and disrupted extracellular matrix (ECM) homeostasis are positive related to the progression of intervertebral disc.** **(a, b)** Immunohistochemical (IHC) staining of aggrecan (Acan), collagen type II (Col2a1), matrix metalloproteinase 13 (Mmp13) and a disintegrin and metalloproteinase with thrombospondin motif 5 (Adamts5) in human NP samples. bar, 50 μm. *N* = 6. **(c, d)** TUNEL staining of human NP samples. bar, 50 μm. *N* = 6. **(e, f)** IHC staining of active Caspase3, Bcl2 and Bax in human NP samples. bar, 50 μm. *N* = 6. Results are expressed as mean ± standard deviation (s.d.). ***P* < 0.01, ****P* < 0.001.


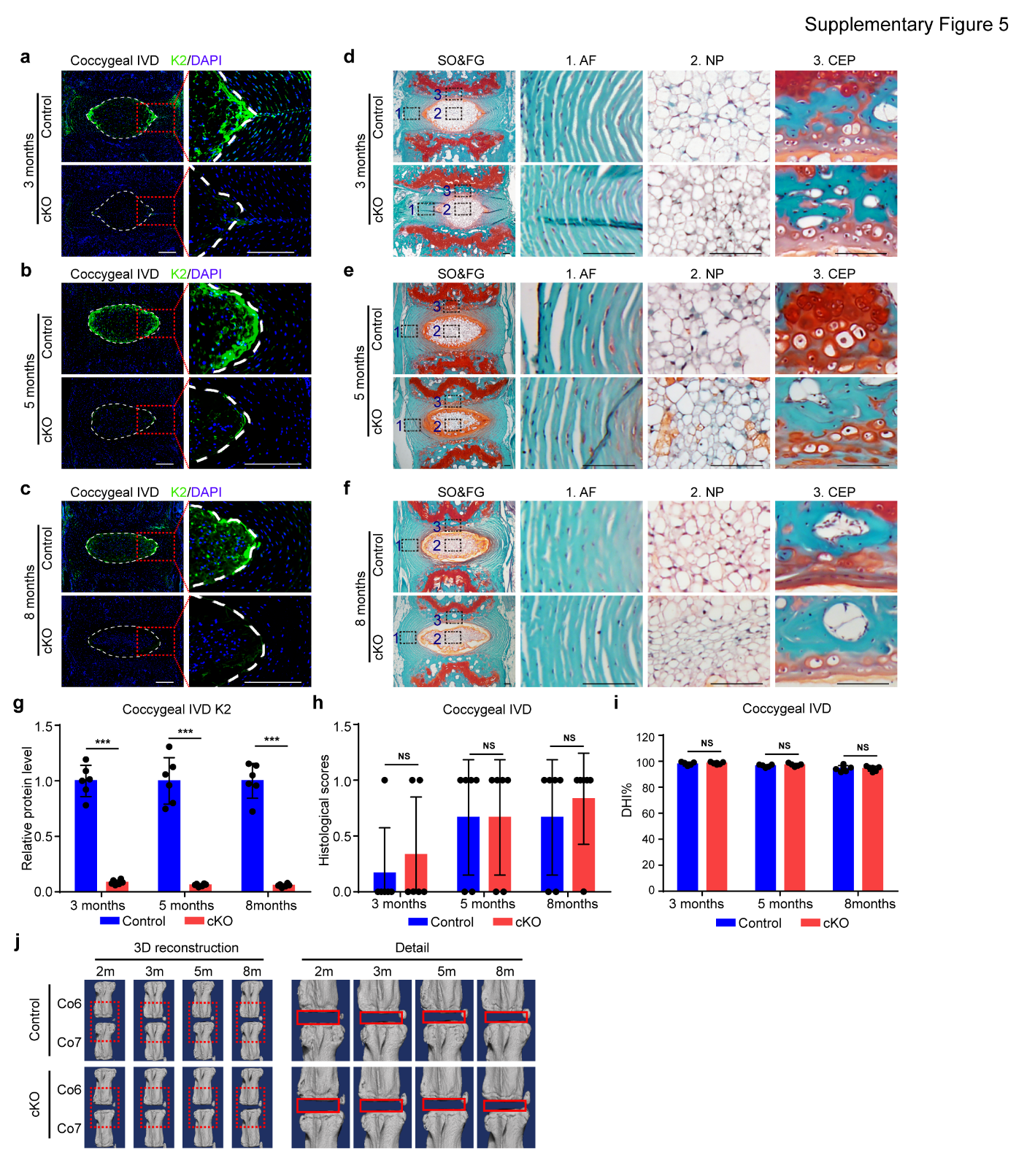


**Supplementary Figure 5.** **Kindlin-2 deletion in coccygeal intervertebral discs.** **(a-c, g)** IF staining of K2 in coccygeal IVDs in control and cKO mice at 3, 5 and 8 months of age. bar, 200 μm. *N* = 6. **(d-f, h)** Safranin O and Fast Green (SO&FG) staining and histological scores of coccygeal IVDs in control and cKO mice at 3, 5 and 8 months of age. bar, 100 μm. *N* = 6. **(i, j)** Disc height index percentage (%DHI) of coccygeal IVDs (Co6-7) of the control and cKO mice was evaluated by μCT. *N* = 6. Results are expressed as mean ± standard deviation (s.d.). NS, no statistical significance, ****P* < 0.001. AF, annulus fibrosus; NP, nucleus pulposus; CEP, cartilaginous endplate.


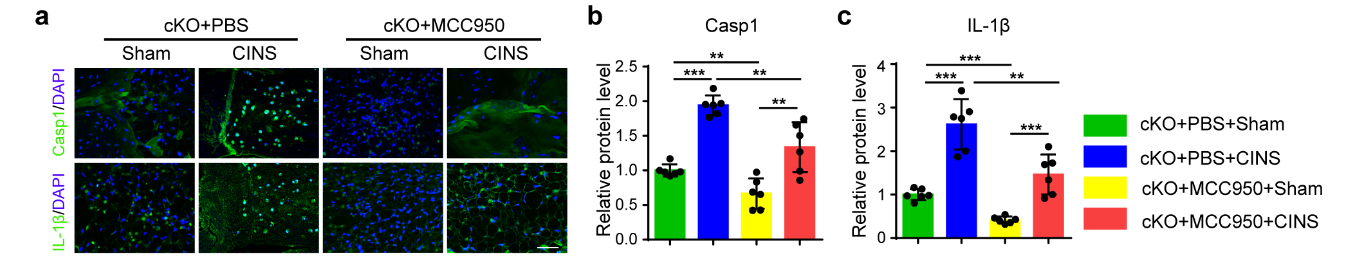


**Supplementary Figure 6.** **The activation of NLRP3 inflammasome was inhibited by MCC950.** **(a-c)** IF staining of caspase-1 (Casp1) and IL-1β in NP tissues in coccygeal IVDs of control and cKO mice, which were treated with or without CINS, and then treated with or without MCC950. bar, 50 μm. *N* = 6. Results are expressed as mean ± standard deviation (s.d.). ***P* < 0.01, ****P* < 0.001.


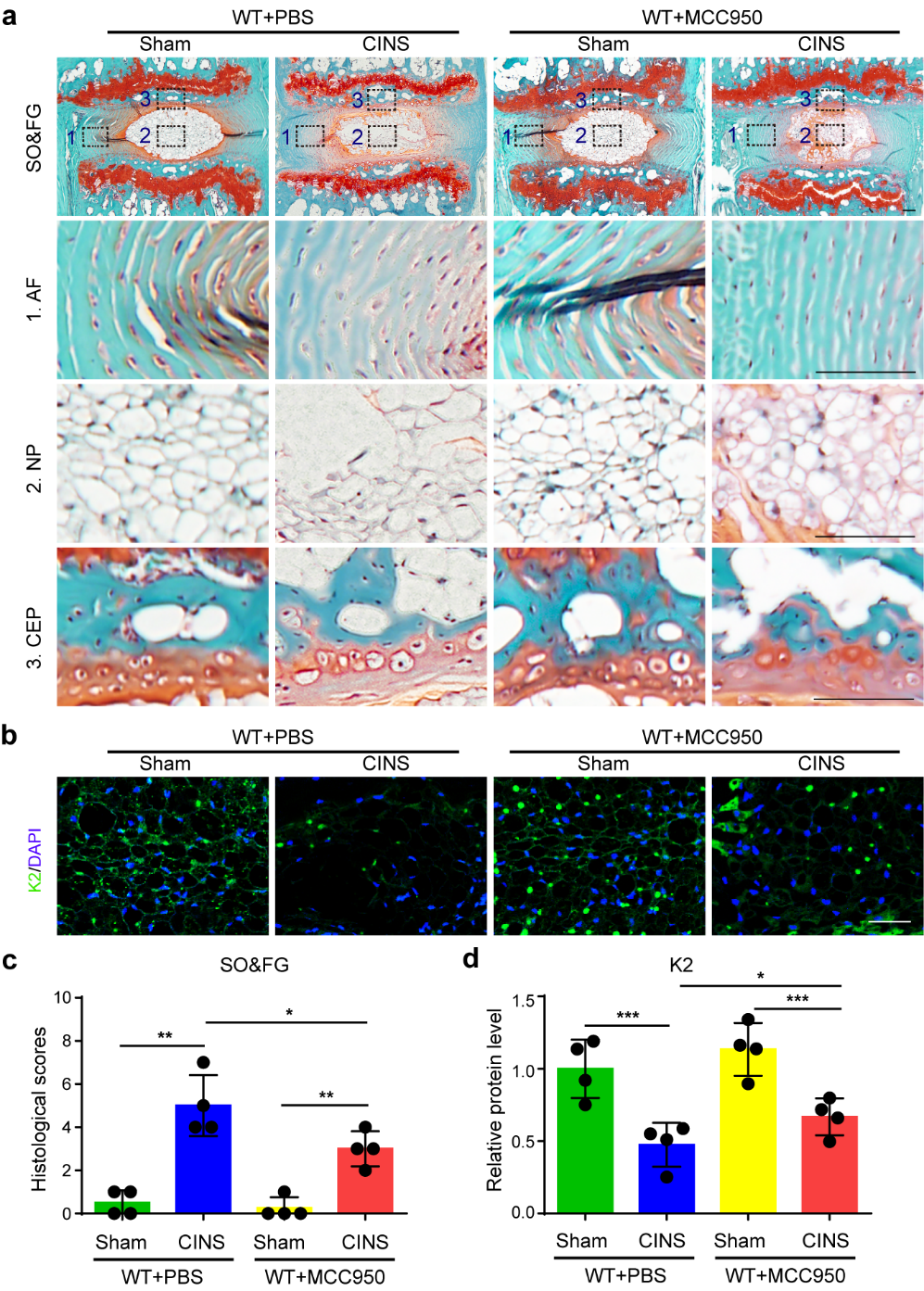


**Supplementary Figure 7.** **Effects of MCC950 treatment on needle injury defects in WT mice.** **(a, c)** SO&FG staining and histological scores of coccygeal IVDs in WT mice. Mice were treated with or without coccygeal IVD needle stab (CINS) at 12 weeks of age, and then intraperitoneally injected with MCC950 at dose of 10 mg/kg body weight or equivalent volume of PBS every 2 days for 6 weeks. Scale bar, 100 μm. *N* = 4. **(b, d)** IF staining of Kindlin-2 in NP tissues in coccygeal IVDs of WT mice treated as in (a). Scale bar, 50 μm. *N* = 4. Results are expressed as mean ± standard deviation (s.d.). **P* < 0.05, ***P* < 0.01, ****P* < 0.001. AF, annulus fibrosus; NP, nucleus pulposus; CEP, cartilaginous endplate.


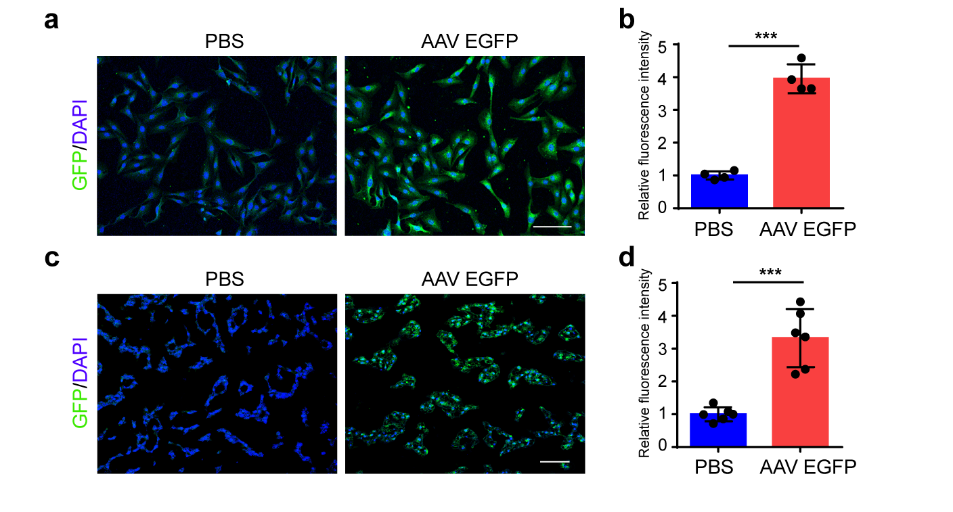


**Supplementary Figure 8.** **GFP signal in human NP cells and rat NP tissues after** **adeno-associated virus infection.** **(a, b)** The GFP signal in human NP cells infected with adeno-associated virus expressing enhanced green fluorescent protein (AAV EGFP) was quantified by relative fluorescence intensity. bar, 100 μm. *N* = 4. **(c, d)** The GFP signal in rat NP tissues at three weeks after AAV EGFP infection was quantified by relative fluorescence intensity. bar, 100 μm. *N* = 6. Results are expressed as mean ± standard deviation (s.d.). **P* < 0.05, ***P* < 0.01, ****P* < 0.001.


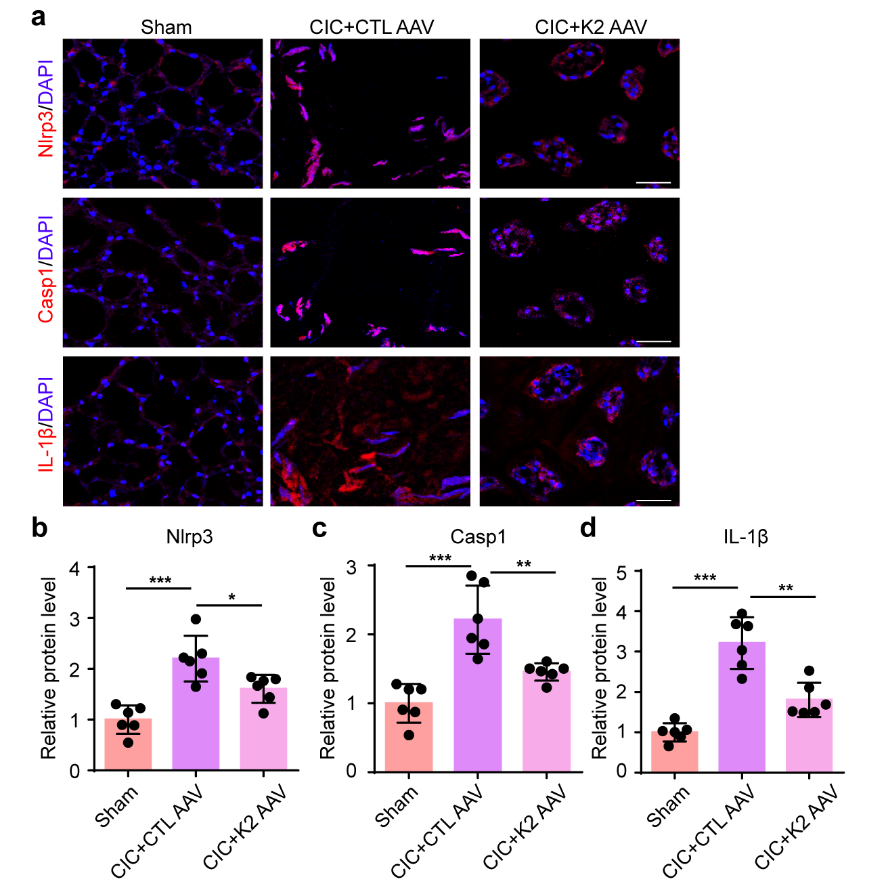


**Supplementary Figure 9.** **Administration of Kindlin-2 adeno-associated virus AAV inhibited the Nlrp3 inflammasome activation.** **(a-d)** IF staining of Nlrp3, Casp1 and IL-1β in NP tissues in rat coccygeal IVDs infected with control adeno-associated virus (CTL AAV) or K2 AAV, and then treated with or without coccygeal IVDs compression (CIC). bar, 50 μm. *N* = 6. Results are expressed as mean ± standard deviation (s.d.). **P* < 0.05, ***P* < 0.01, ****P* < 0.001.


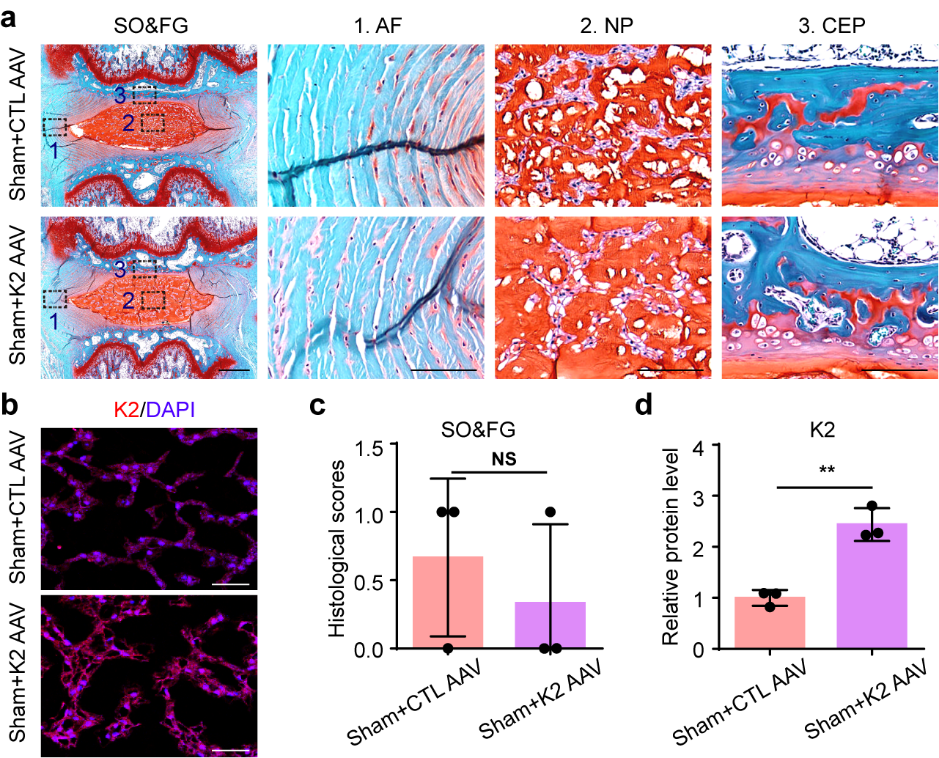


**Supplementary Figure 10.** **Effect of AAV-mediated Kindlin-2 overexpression on unloaded IVD structure in rats.** **(a, c)** SO&FG staining and histological scores of coccygeal IVDs in rats. Rats were treated with control adeno-associated virus (CTL AAV) or K2 AAV (3.2 X 10^10^ particles in 2 μL) by direct injection into the coccygeal IVDs at 3 months of age, and suffered sham surgery three weeks later, then sacrificed at two weeks after the sham surgery. Scale bar, 500 or 100 μm. *N* = 3. **(b, d)** IF staining of Kindlin-2 in NP tissues in rat coccygeal IVDs infected with CTL AAV or K2 AAV. bar, 50 μm. *N* = 3. Results are expressed as mean ± standard deviation (s.d.). NS, no statistical significance, ***P* < 0.01. AF, annulus fibrosus; NP, nucleus pulposus; CEP, cartilaginous endplate.

**Supplementary Tables**

**Supplementary Table 1.** Characteristics details of the patients enrolled in the study.

| Case no. | Age (years) | Gender | Diagnosis | Disc level | Pfirrmann grading |
| --- | --- | --- | --- | --- | --- |
| Case 1 | 28 | M | Lumbar disc herniation | L4/5 | II |
| Case 2 | 35 | F | Lumbar disc herniation | L4/5 | II |
| Case 3 | 42 | M | Lumbar disc herniation | L5/S1 | II |
| Case 4 | 75 | M | Lumbar disc herniation | L4/5 | III |
| Case 5 | 48 | F | Lumbar disc herniation | L4/5 | III |
| Case 6 | 29 | M | Lumbar disc herniation | L4/5 | III |
| Case 7 | 44 | F | Lumbar disc herniation | L5/S1 | III |
| Case 8 | 63 | M | Lumbar disc herniation | L4/5 | III |
| Case 9 | 27 | M | Lumbar disc herniation | L4/5 | III |
| Case 10 | 30 | M | Lumbar disc herniation | L4/5 | IV |
| Case 11 | 61 | F | Lumbar disc herniation | L4/5 | IV |
| Case 12 | 52 | F | Lumbar disc herniation | L5/S1 | IV |
| Case 13 | 28 | F | Lumbar disc herniation | L4/5 | IV |
| Case 14 | 43 | M | Lumbar disc herniation | L4/5 | IV |
| Case 15 | 62 | M | Lumbar disc herniation | L5/S1 | V |
| Case 16 | 82 | F | Lumbar disc herniation | L5/S1 | V |
| Case 17 | 56 | F | Lumbar disc herniation | L4/5 | V |
| Case 18 | 57 | M | Lumbar disc herniation | L4/5 | V |

**Supplementary Table 2.** Primer sequences used in PCR genotyping and siRNA sequences used in siRNA transfection

| **Gene** | **Forward (5’-3’)** | **Reverse (5’-3’)** |
| --- | --- | --- |
| Kindlin-2 | TGTGTTTCAAAGGTACTGGTCA | ACAATGGTGCTTTGCCTACA |
| Cre | GATCTCCGGTATTGAAACTCCAGC | GCTAAACATGCTTCATCGTCGG |
|  | | |
| **siRNA** | **Sense (5’-3’)** | **Antisense (5’-3’)** |
| NC | UUCUCCGAACGUGUCACGUTT | ACGUGACACGUUCGGAGAATT |
| K2 #1 | GCAUCCAGGCAGACGCCAATT | UUGGCGUCUGCCUGGAUGCTT |
| K2 #2 | GCCGGUAACAUCACCAGAATT | UUCUGGUGAUGUUACCGGCTT |
| K2 #3 | GCCUCAAGCUCUUCUUGAUTT | AUCAAGAAGAGCUUGAGGCTT |

**Supplementary Table 3.** Antibody information.

| Antibody | Company | Catalog # | Application/Dilution |
| --- | --- | --- | --- |
| Kindlin-1 | Sigma-Aldrich | SAB4200465 | IF (1:100) |
| Kindlin-2 | Proteintech | 11453-1-AP | WB (1:1000); IF (1:100) |
| Kindlin-3 | CST | 13843 | IF (1:100) |
| Talin | Abcam | Ab110080 | IF (1:200) |
| Vinculin | Sant Cruz | sc-73614 | IF (1:200) |
| Aggrecan | Abcam | ab36861 | WB (1:1000); IHC (1:100); IF (1:100) |
| Aggrecan | ABclonal | A8536 | WB (1:1000) |
| Col2a1 | ABclonal | A1560 | WB (1:1000) |
| Col2a1 | Sant Cruz | sc-52658 | IHC (1:100); IF (1:100) |
| Mmp13 | Abcam | ab39012 | WB (1:1000); IHC (1:100); IF (1:100) |
| Adamts5 | Abcam | ab41037 | WB (1:500); IHC (1:50); IF (1:50) |
| Active caspase 3 | Sigma-Aldrich | C8487 | WB (1:500); IHC (1:100); IF (1:100) |
| Bcl2 | BOSTER | A00040 | WB (1:1000); IHC (1:100); IF (1:100) |
| Bax | CST | 2772 | WB (1:1000); IHC (1:100); IF (1:100) |
| Nlrp3 | BOSTER | BA3677 | WB (1:1000); IHC (1:200); IF (1:200) |
| Caspase-1 | ABclonal | A0964 | WB (1:1000); IHC (1:200); IF (1:200) |
| IL-1β | ABclonal | A1112 | WB (1:1000); IHC (1:200); IF (1:200) |
| Gapdh | Proteintech | 60004-1-Ig | WB (1:3000) |
